# Cost-Effectiveness Study of Three Antimalarial Drug Combinations in Tanzania

**DOI:** 10.1371/journal.pmed.0030373

**Published:** 2006-10-10

**Authors:** Virginia Wiseman, Michelle Kim, Theonest K Mutabingwa, Christopher J. M Whitty

**Affiliations:** 1 Department of Public Health and Policy, London School of Hygiene and Tropical Medicine, London, United Kingdom; 2 National Institute for Medical Research, Dar-es-Salaam, Tanzania; 3 Department of Infectious and Tropical Diseases, London School of Hygiene and Tropical Medicine, London, United Kingdom; Rigshospitalet, Denmark

## Abstract

**Background:**

As a result of rising levels of drug resistance to conventional monotherapy, the World Health Organization (WHO) and other international organisations have recommended that malaria endemic countries move to combination therapy, ideally with artemisinin-based combinations (ACTs). Cost is a major barrier to deployment. There is little evidence from field trials on the cost-effectiveness of these new combinations.

**Methods and Findings:**

An economic evaluation of drug combinations was designed around a randomised effectiveness trial of combinations recommended by the WHO, used to treat Tanzanian children with non-severe slide-proven malaria. Drug combinations were: amodiaquine (AQ), AQ with sulfadoxine-pyrimethamine (AQ+SP), AQ with artesunate (AQ+AS), and artemether-lumefantrine (AL) in a six-dose regimen. Effectiveness was measured in terms of resource savings and cases of malaria averted (based on parasitological failure rates at days 14 and 28). All costs to providers and to patients and their families were estimated and uncertain variables were subjected to univariate sensitivity analysis. Incremental analysis comparing each combination to monotherapy (AQ) revealed that from a societal perspective AL was most cost-effective at day 14. At day 28 the difference between AL and AQ+AS was negligible; both resulted in a gross savings of approximately US$1.70 or a net saving of US$22.40 per case averted. Varying the accuracy of diagnosis and the subsistence wage rate used to value unpaid work had a significant effect on the number of cases averted and on programme costs, respectively, but this did not change the finding that AL and AQ+AS dominate monotherapy.

**Conclusions:**

In an area of high drug resistance, there is evidence that AL and AQ+AS are the most cost-effective drugs despite being the most expensive, because they are significantly more effective than other options and therefore reduce the need for further treatment. This is not necessarily the case in parts of Africa where recrudescence following SP and AQ treatment (and their combination) is lower so that the relative advantage of ACTs is smaller, or where diagnostic services are not accurate and as a result much of the drug goes to those who do not have malaria.

## Abstract 2

A randomised effectiveness trial of antimalarial drug combinations used to treat Tanzanian children found artemether-lumefantrine to be the most cost-effective.

## Editors' Summary

**Background.:**

For many years, malaria was treated with a course of a single drug. This type of treatment made it easy for malaria parasites to become resistant to antimalarial drugs. This is a major factor contributing to the continuing high death rate from the disease. However, although parasites can easily adapt to resist one drug, adapting to combinations of two or three drugs is much harder. Scientists have therefore developed combinations of antimalarial drugs. One component of these combinations is artemisinin—derived from a Chinese shrub. However, these combination therapies are much more expensive than the older treatments.

The regions worst affected by malaria—Africa and Asia—are also the poorest. And, in these areas, where both individual and government resources are scarce, antimalarial treatments must be cost-effective as well as clinically effective.

**Why Was This Study Done?:**

Most of the estimated 1 million to 3 million people worldwide killed by malaria every year are young children in sub-Saharan Africa. Growing drug resistance, poor prevention programs, and a frequent inability of patients to pay for treatment mean that effective therapy is desperately needed in this part of the world. However, because of differences in drug resistance between regions, a drug combination will not work everywhere. In addition, because of low annual incomes (the average in Tanzania is US$120), heavy subsidies will probably be required to ensure that combination treatments are widely used. With several healthcare problems competing for resources, policymakers are likely to subsidize only the most cost-effective treatments. The researchers wanted to provide policymakers with information on how different combinations of malaria drugs compare in terms of costs, health effects, and cost-effectiveness, so that they can decide which treatment is best for their region.

**What Did the Researchers Do and Find?:**

They compared three combinations that the World Health Organization recommends for countries when making the transition from single-drug therapy. The three combinations—amodiaquine (AQ) and sulfadoxine-pyrimethamine (SP); AQ and artesunate (AS); and artemether-lumefantrine (AL)—were used to treat Tanzanian children. The researchers wanted to find out how many cases of malaria each combination averted (which is also an indication of how much money it saved) and how much the treatment cost. They looked at costs and savings from the perspectives of both healthcare providers and patients.

Compared with no treatment, AL proved to be the most cost-effective; although it cost more for the provider (US$3.01 at day 28 of treatment) than the others, its effectiveness in getting rid of the parasite meant it would save the cost of future treatment. By day 28, AL had averted 382 cases of malaria compared with 279 for AQ+AS, 181 for AQ+SP, and 57 for AQ alone. Also, higher proportions of inaccurate diagnoses of malaria led to lower cost-effectiveness of treatments.

**What Do These Findings Mean?:**

Despite being more expensive, newer drugs can be cost-effective where alternatives fail. Although AL was the most cost-effective in places (such as Tanzania) where the malaria parasites are highly resistant to SP and AQ, the picture is likely to change for other areas. In West Africa, for example, AQ resistance is lower, and AQ+SP and AQ+AS would probably be more cost-effective. And in areas where both these combinations are just as good as AL in preventing recurring disease, they would be more cost-effective than AL. However, since AQ and SP have been used singly for many years, the likelihood is that resistance to these drugs will continue to increase. Accurate diagnosis turns out to be very important for maintaining the cost-effectiveness of combination antimalarial therapies. This will be essential if they are to be incorporated as a sustainable part of local health policies. The researchers also point out that, depending on which perspective is taken (provider or patient), the cost-effectiveness of treatments differs, making it important to compare like with like.

Although investing in costly AL treatments and improving diagnostic capabilities will be a challenge for African governments that currently spend less than US$5 per person per year on healthcare, it will be necessary if they are to seriously tackle the malaria epidemic.

**Additional Information.:**

Please access these Web sites via the online version of this summary at http://dx.doi.org/10.1371/journal.pmed.0030373

- The US enters for Disease Control and Prevention provides malaria information aimed at the general public, physicians, and health workers
- The Wellcome Trust; also has malaria information for the general public and covers the science of malaria research, including a downloadable animation of the parasite's life cycle
- Medicines for Malaria Venture (MMV) is a charity created to develop new antimalarial drugs through public-private partnerships

## Introduction

Rising drug resistance levels to conventional monotherapy has resulted in strong arguments for a move to combination treatment for all malaria in Africa, especially artemisinin-based combination therapies (ACTs) [1–2]. The relatively high cost of these new medicines compared to current monotherapy represents a major barrier to their effective implementation [3–6]. Currently most people pay for their own malaria treatment, but whilst in areas where monotherapy is failing they are prepared to pay more for combination therapy than for monotherapy, they are not prepared to pay the current market cost of ACTs [6]. This means that subsidising these drugs is likely to be the only realistic option if they are to reach those who need them most, and especially if they are to reach the poorest [7]. In Africa, where there are many urgent competing priorities, this policy is unlikely to be either sustainable or politically realistic unless it is cost-effective. Theoretical projections of the cost-effectiveness at a societal level have suggested that it is likely to be a reasonably cost-effective approach compared to other public health interventions [8].

Studies have not examined the cost-effectiveness of these drugs in areas of Africa with failing monotherapy in using original data, and in particular we are not aware of any that have compared the current treatment options available to public health and clinical decision-makers in Africa. These are essential data for rational policy-making if public funds (e.g., a subsidy) are likely to be involved. We therefore set out to compare the cost-effectiveness of the three currently available drug combinations to treat children with non-severe, slide-proven malaria compared to monotherapy (amodiaquine [AQ]). The combinations were AQ with sulfadoxine-pyrimethamine (AQ+SP), AQ with artesunate (AQ+AS) or artemether-lumefantrine ([AL], six-dose regimen). The study was undertaken in the context of a clinical trial where the effectiveness of the drugs taken unsupervised was measured directly [9]. In this study we compare the costs, health effects, and cost-effectiveness of each treatment.

## Methods

The study was conducted at Teule Hospital, a designated district hospital located in Muheza, a small town in the northeast district of Tanga, Tanzania with an EIR (entomological inoculation rate) of over 300 and where the average individual has around three clinical attacks of malaria a year [10]. There are high levels of drug resistance to both SP and amodiaquine monotherapy [9,11,12]. The clinical effectiveness of drugs and combinations was determined by way of a trial conducted between September 2002 and October 2004, reported elsewhere [9]. In brief, 1,811 children were randomised to one of the four treatment arms. By day 28, the parasitological failure rates were 76% for AQ monotherapy, 61% for AQ+SP, 40% for AQ+AS, and 21% for AL.

An incremental approach was used to estimate costs and effects. This involved comparing each of the new combination medicines against the existing monotherapy of AQ. SP is currently first-line and AQ second-line treatment for uncomplicated malaria in Tanzania. Pilot testing for this trial revealed unacceptably high levels of resistance to SP, so AQ was selected as the monotherapy against which each combination medicine was compared. Economic costs were estimated using the bottom-up approach in which all resources required in the delivery of treatment are valued [13]. Health effects were measured in terms of cases of malaria averted (based on parasitological failure rates at days 14 and 28); details of the methodology of the effectiveness trial are published [9].

Findings are presented from both a provider (hospital) and a societal perspective. For the provider perspective, only those costs and effects borne by the provider are considered; this is the cost-effectiveness from the point of view of a ministry of health. For the societal perspective, the costs and effects borne by patients and their families are combined with those of the provider, which is especially relevant when considering the case for subsidy.

The main areas of resource use for the health service were medication, personnel, rent for hospital space, and use of a microscope. Published market drug prices were used [14–16], and where a child's dose was not specified, it was assumed to be 50% of an adult dose. Staff salaries and building costs were prorated based on the number of malaria patients under the age of 5. The microscope was annualised over an estimated useful life of two years at a discount rate of 3% [17].

Families of patients incurred both indirect and direct costs. Direct costs included out-of-pocket expenses such as hospital fees, transport, and medication, as well as miscellaneous expenditures such as informal payments made to medical staff, food, and blankets. Hospital fees were not charged for children under the age of 5, but fees were rendered if the registration card needed to be replaced. Medical costs included extra antibiotics or blood transfusions. Indirect costs included time lost as a result of caring for a sick child at home, travelling to hospital, and waiting at hospital for treatment. Time lost was valued at the prevailing minimum subsistence wage rate in Tanzania (i.e., 30,000 Tanzanian shillings in 2004).

Estimation of resources savings were based on the cost of current first-line treatment with SP. Current therapy differed from the intervention treatments in two key respects: type of medicine used and the time families spent seeking treatment. Current treatment with SP was valued using the hospital's procurement prices at the dispensary. Indirect costs were measured for patients taking the current treatment. These costs were the same as for AQ, as parasitological failure rates and drug cost are almost exactly the same as for SP in this setting [9]. For the remaining combination treatments, indirect costs were assumed to be lower due to increased levels of effectiveness. Rates of relative effectiveness for each of the combinations have been applied to the indirect costs of current treatment.

Costs incurred at the household level were collected through structured interviews at the hospital outpatient facility. Sixty-three parents of febrile children under the age of 5 diagnosed with malaria were selected between March and April 2005. Parents were purposively selected from different ethnic, socioeconomic, and rural/urban locations. All participants gave informed consent. All remaining costs were collected from the principle investigators of the trial and from hospital accounting records. Staff salaries were obtained from the standard payroll scales for Tanzania. All costs were converted from Tanzanian shillings to US dollars based on the exchange rate prevailing at the outset of the trial (i.e., 1,076 Tzs = US$1) and discounted at 3% per year to estimate their present value [18]. Research costs were excluded from the analysis. Uncertain variables (where variation in the assumptions might affect cost-effectiveness) were subjected to a univariate sensitivity analysis including accuracy of diagnosis, drug prices, the discount rate, and subsistence wage. The study was given clearance by the ethics committees if the National Institute for Medical Research, Tanzania and the London School of Hygiene & Tropical Medicine.

## Results

The programme costs for each treatment are shown in Table 1. For the provider, the higher unit price of AL meant that the cost of implementing this treatment was higher than for the others. For patients and their families, current treatment with SP and treatment with AQ were the most costly, due to the need for retreatment. The indirect costs to patients and their families were the largest component of programme costs for all treatments.

**Table 1:**
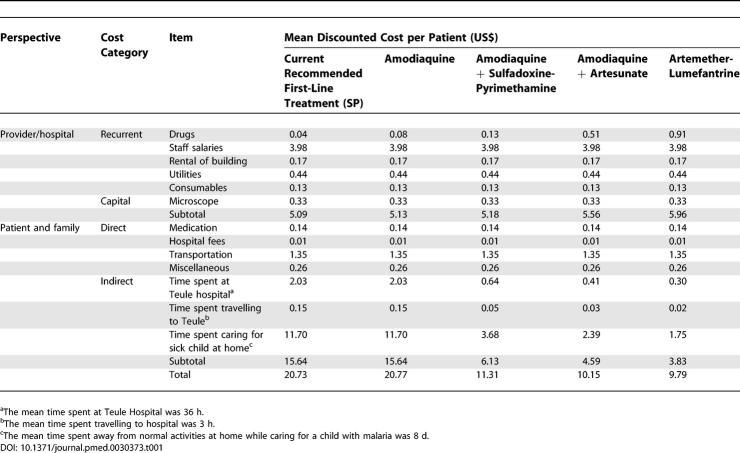
Programme Costs

Cost-effectiveness data are shown in Table 2. By day 14, AL was revealed to be the most effective treatment with 495 cases averted. By day 28 the effectiveness of all treatments had fallen but AL remained most effective, averting 382 cases of malaria. Comparing cost-effectiveness ratios for the three combination treatments against monotherapy (AQ) revealed that from a societal perspective AL was most cost-effective at day 14; resulting in a gross saving of US$1.51 or a net saving of US$22.24 per case averted. At day 28 the difference between the two most cost-effective treatments of AL and AQ+AS was negligible; both resulted in gross savings of approximately US$1.70 or a net saving of US$22.40 per case averted. From a provider perspective, AL was most costeffective at days 14 and 28.

**Table 2:**
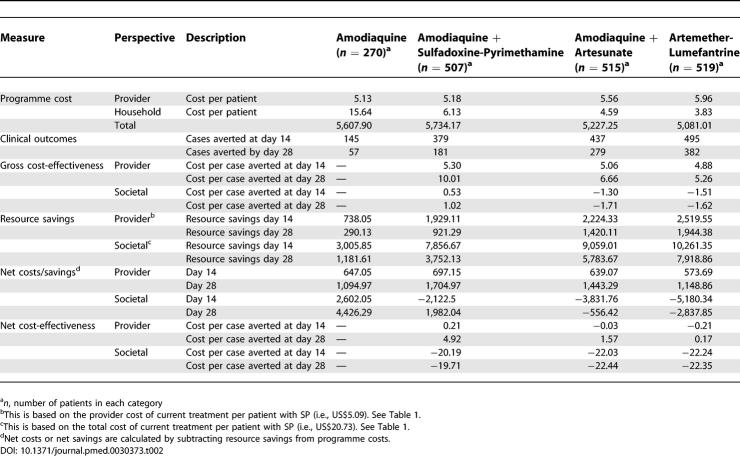
Costs (US$), Effects, and Cost-Effectiveness

The greatest resource savings were associated with AL. From a societal perspective, total savings were US$10,261 at day 14 and US$7,919 at day 28. The equivalent figures from a provider perspective are US$2,520 at day 14 and US$1,944 at day 28. For all treatments, savings were higher at day 14 compared with day 28. The greatest reduction in savings to society between days 14 and 28 was reported for AQ (61%). Comparing the three combination treatments against monotherapy (AQ) showed that from a societal perspective AL was again most-cost-effective at day 14. By day 28 there was only a minor difference between AL and AQ+AS.

The results of the sensitivity analysis are shown in Table 3. The effectiveness of all treatments was strongly influenced by accuracy of diagnosis. For example, assuming that 70% of all patients treated for malaria do not in fact have malaria, the number of incremental cases averted using AL falls from 325 to 98 at day 28 while programme costs remain the same. Each combination treatment is compared to monotherapy, which is also assumed to experience a 70% reduction in accuracy of diagnosis. So while cost-effectiveness falls, combinations such as AL remain better value for money *relative* to monotherapy by saving more resources and averting more cases of malaria. Changing the subsistence wage rate also led to notable changes in costs, but this, again, did not change the fact that combination treatments such as AL continued to be more cost-effective than monotherapy. Altering drug prices or the discount rate had little effect on the findings of this study.

**Table 3:**
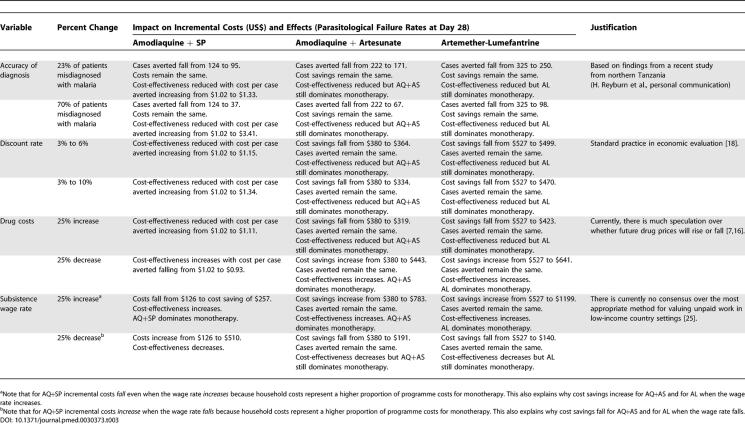
Univariate Sensitivity Analysis

## Discussion

This is, to our knowledge, the first field-based study to compare the cost-effectiveness of the three available drug combinations recommended by a consultative group of WHO for deployment by countries when they move from monotherapy [2]. For each treatment, two sets of cost-effectiveness ratios were presented. The first set were gross estimates that did not take into account potential resource savings; the second set did (i.e., net cost-effectiveness ratios). Both sets of ratios indicate that, whilst AL is the most expensive drug, in this context of malaria that is highly resistant to both SP and AQ, it is the most-cost-effective of the four malaria treatments. Despite the drop in the effectiveness of all treatments between days 14 and 28 due to a combination of reinfection and recrudescence of malaria, savings at day 28 for AL are one-third higher than the initial programme costs faced by providers and patients.

This clear and striking finding has to be interpreted with three notes of caution, all of which are important in trying to generalise the messages of the study. Firstly, and obviously, AL is the most cost-effective drug despite being the most expensive because it is significantly more effective than other options and is therefore likely to reduce the need for further treatment. This will not, however, necessarily be the case in parts of Africa where recrudescence following SP and AQ treatment (and their combination) is lower, so the relative advantage of this ACT is smaller [19,20]. In particular, where rates of AQ resistance are lower—which they are especially in West Africa—both AQ+AS and AQ+SP will be relatively more cost-effective, and where these combinations are as clinically effective as AL in preventing recrudescence they will be more cost-effective than AL.

Diagnostic accuracy has important operational implications. Our results show that accuracy of diagnosis plays an important role in the estimation of effectiveness of combination therapy for malaria. If cost-effectiveness of ACTs is undermined in this way then political support for them is unlikely to be sustained. In this study, double-read research microscopy was used to confirm malaria and only those with a positive result received treatment. In practice, however, microscopes are not widely used in many parts of Africa and clinical diagnosis tends to be the norm. Moreover, recent studies in Tanzania and elsewhere provide evidence that, in practice, many patients with a negative result go on to be treated for malaria [21]. Drawing on local data demonstrating that between 23% and 70% of all those with a negative test will in fact, be given an antimalarial (H. Reyburn, personal communication), we explored the impact on cost-effectiveness of these misdiagnosis rates. If approximately 70% of patients are wrongly diagnosed with malaria (and in low-transmission settings the proportion can be even higher), then the cost per case averted for AQ+SP compared to AQ rise three-fold. AL and AQ+AS remain dominant over AQ but the economic case for these treatments is weakened since cases averted have fallen and cost savings remain the same. Given the importance of accuracy of diagnosis, we have disaggregated all cost and effects so that cost-effectiveness ratios can be easily recalculated to take account of different levels of diagnostic accuracy. Improving diagnostic accuracy becomes a critical issue if ACTs are to be made more cost-effective and their deployment is to be sustainable.

While resource savings are a legitimate outcome of many interventions they tend inevitably to be less precise than estimates of programme costs. For example, adherence levels may be higher under trial conditions. The data from this study come from an effectiveness study conducted in a setting similar to outpatient practice in children, with drugs taken unsupervised at home, rather than efficacy data from trials where all doses are supervised, but families may have perceived trial-based treatment to be of a higher quality and this may promote adherence and, in turn, cases of malaria averted. The lack of payment for ACTs in the trial reflects the current policy in Tanzania. However, the influx of these drugs in the private sector may give families an incentive to save or to sell extra drugs instead of taking the correct dose [9]. These downward pressures on resource savings will to some extent be countered by other factors such as savings to patients and their families resulting from spending less time travelling to hospital, waiting at hospital for treatment, or caring for sick children at home.

In this study all cost and cost-effectiveness estimates are shown from two perspectives: societal and provider. Depending on the perspective taken the results differ substantially. For example, both the programme costs and the resource savings to patients and their families are greater than those to the health service. This translates into lower cost-effectiveness ratios under a societal perspective than under a provider one. When comparing these results with cost-effectiveness ratios for other malaria control or health interventions it is important to ensure that, as far as possible, like is being compared with like and the starting point for this should be the perspective of the evaluation. Again, we have shown estimates based on both perspectives to allow readers to adopt the one that most suits the context in which they are working.

Finally, it has been suggested that a cost-effectiveness ratio less than double the annual income per capita might be an acceptable threshold value for most governments deciding which intervention to fund [22]. The findings from this study fall well below the average annual per capita income of US$120 for Tanzania [23]. “Thresholds,” however, are only a guide to decision-makers. Medicines such as AL require considerable upfront investment in order to realise any benefits, and in particular are likely to need investment in improving diagnostic accuracy. It is difficult to see how most African governments can meet this challenge alone given that they currently devote less than US$5 per person annually to public health [24]. This study demonstrates that in areas of Africa where monotherapy is failing badly, ACTs, despite being more expensive than monotherapy, can be very cost-effective provided that the drugs are prescribed only to those with malaria. The external investment, which will be needed to subsidise drugs even at current costs, and to improve targeting to those with malaria, is clearly justified on the basis of these data.

## Abbreviations

ACT: artemisinin-based combination therapy
AL: artemether-lumefantrine
AQ: amodiaquine
AS: artesunate
SP: sulfadoxine-pyrimethamine
